# Supplementary material for: Benchmarking Diffusion Annealing-Based Bayesian Inverse Problem Solvers
Source: IEEE Open J Signal Process. Author manuscript; Available in PMC 2025 Sep 24. (PMC12456369; doi:10.1109/ojsp.2025.3597867)
Supplement: supp1-3597867 [file NIHMS2106639-supplement-supp1-3597867.pdf]

.

Supplementary Material For “Benchmarking Diffusion Annealing-Based Bayesian Inverse Problem Solvers”

Evan Scope Crafts[1], Member, IEEE, and Umberto Villa[1, 2], Member, IEEE

[1]Oden Institute for Computational Engineering and Sciences, The University of Texas at Austin, Austin, TX USA 78712
[2]Dept of Biomedical Engineering, The University of Texas at Austin, Austin, TX USA 78712

This document contains additional visualizations from the numerical studies described in the main paper, as well as a proof-of-concept results demonstrating the viability of our framework in the context of imaging problems.

I. Additional Visualizations

In this section, we show violin plots [1] of the central moment discrepancy (CMD) and maximum mean discrepancy (MMD) errors to provide additional insight into how the BIPSDA algorithms perform across the posterior sampling trials. The ground-truth and BIPSDA algorithm posterior samples for all four studies are also provided at: https://doi.org/10.7910/DVN/0L5KGB to facilitate further analysis of the data.

Figure S1 shows two-sided violin plots of the CMD and MMD errors across posterior sampling trials for the stylized inpainting study in the low noise regime. Note that in these violin plots, and all subsequent violin plots, the densities have been normalized to have unit width for ease of visualization. As can be seen, the learned score model is not a major source of error in the posterior sampling. Further, while all BIPSDA algorithms perform well on this problem, the ‘Lang-ODE’, ‘RTO-TU’, and ‘RTO-TC’ (with analytic score) achieve the strongest performance of the algorithms we tested.

Figure S2 shows the corresponding plots in the high noise regime. As can be seen, the Langevin-based approaches perform poorly across the trials. The RTO-based approaches perform well with respect to both the CMD and MMD metrics, but have high variance across the trials—there is a two-order-of-magnitude difference in performance between the best-performing and worst-performing trials. The MAP based approaches are competitive with the RTO based approaches with respect to the CMD metric but perform significantly worse with respect to the MMD metric. This is not a surprising result, as the MAP based approaches underestimate the variance of the posterior modes and the MMD metric is very sensitive to the local structure of the distributions.

Figure S3 displays the violin plots corresponding to the stylized x-ray tomography study. As can be seen, while all approaches we tested perform competitively on this problem, the ‘RTO-TU’ and ‘RTO-TC’ (with analytic score) approaches perform the best across the trials and are capable of achieving very high accuracy. We also note that ‘RTO-ODE’ approach performs significantly worse than the ‘RTO-TU’ and ‘RTO-TC’ approaches. This result, which is consistent with observations in the other three studies we conducted, suggests that the ‘TU’ and ‘TC’ techniques are better suited for use with the RTO sampler in our framework.

Figure S4 shows the violin plots for the phase retrieval study. As can be seen, the performance of all of the BIPSDA algorithms we tested varies significantly across the trials. While the algorithms are all capable of achieving strong performance on some of the trials, they also all struggle to perform well on the more difficult trials. Specifically, the algorithms struggle to perform well when the posterior is multi-modal and the modes are highly separated. Additional work is therefore required to achieve strong performance on this challenging problem.

II. Imaging Experiments

This section displays proof-of-concept results from applying BIPSDA algorithms to a high-dimensional imaging application. In particular, we applied BIPSDA algorithms to posterior sampling from the box imaging inpainting problem considered in [2], with the prior distribution samples given by the FFHQ dataset and a linear-Gaussian likelihood with forward operator

This work is licensed under a Creative Commons Attribution 4.0 License. For more information, see https://creativecommons.org/licenses/by/4.0/

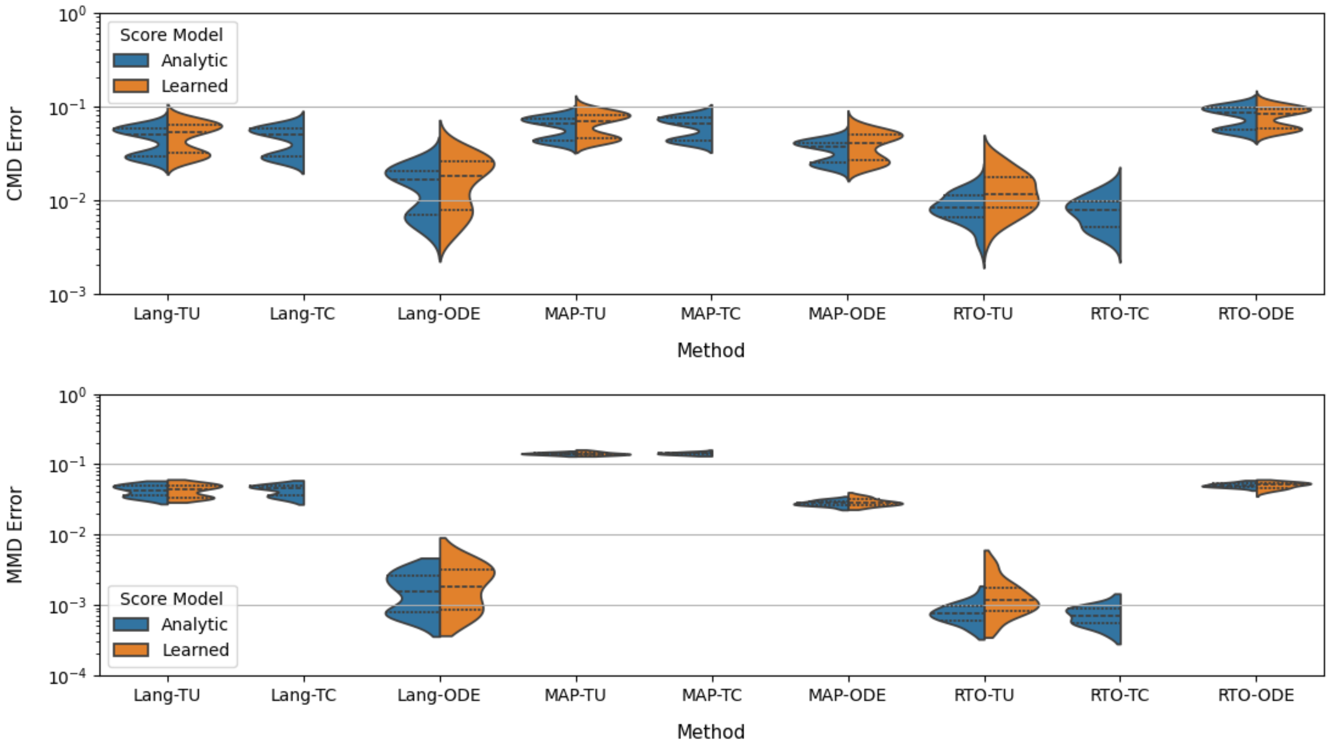

FIGURE S1. Stylized inpainting study, low noise regime: Two-sided violin plots of the CMD errors (top) and MMD errors (bottom) across the one hundred trials. Note that the violin kernel densities in this and all subsequent plots were normalized to have unit width for ease of visualization. As can be seen, the 'Lang-ODE', 'RTO-TU', and 'RTO-TC' (in analytic score setting) approaches are the best performing with respect to both the CMD and MMD metrics.

given by a binary sampling mask. The noise standard deviation was set to 0.05. We refer to [2] for further details of the problem setup and of the pretrained diffusion model. For all methods, the sequence of decreasing timesteps $[t_{N_A}, \cdots, t_0]$ for the annealing loop was chosen as in [2] and $\beta(t) = \sigma(t)/3$ was used in the denoising distribution approximation $\mathbf{C}_{\text{aprx}} = \beta(t)^2\mathbf{I}$. The Langevin dynamics-based sampler for the 'Lang' approaches and the ODE solver for the 'ODE' approaches were also set as in [2]. Finally, the MAP problem arising in the 'MAP' and 'RTO' variants was solved analytically in closed form.

Computational times to generate 100 samples using the proposed methods are given in Table S1. The reported computational times are the average of 10 independent trials. As can be seen, the 'Lang-ODE' algorithm, which corresponds to DAPS [2], is the most expensive of all of the BIPSDA algorithms we tested. This is due to the fact that that the cost of approximating the denoising distribution is dominant in the image inpainting problem setting. The 'ODE' algorithm variants, which require more score model evaluations than the 'TU' variants, are therefore significantly more computationally expensive. Furthermore, the 'MAP' and 'RTO' variants are also significantly faster than 'Lang'.

Uncurated samples from the 'Lang-TU', 'MAP-TU', and 'RTO-TU' algorithms in this problem setting are shown in Figure S5; the results with the 'ODE' algorithm variants are similar and are omitted for ease of presentation. As can be seen, all of the algorithms we tested are capable of producing high-quality and diverse samples for this inverse problem. However, given the lack of analytic ground-truth prior and corresponding lack of ground-truth posterior samples, the main purpose of these results is to showcase the computational feasibility of applying algorithms from the BIPSDA framework to large scale imaging problems, and not to assess the ability of the BIPSDA algorithms to capture the posterior structure. Further work and well characterized benchmark problems are therefore needed to rigorously validate the performance of both BIPSDA algorithms and other diffusion-based posterior sampling algorithms in high-dimensional problem settings.

REFERENCES

[1] J. L. Hintze and R. D. Nelson, "Violin plots: A box plot-density trace synergism," *Am. Stat.*, vol. 52, no. 2, pp. 181–184, 1998.

[2] B. Zhang, W. Chu, J. Berner, C. Meng, A. Anandkumar, and Y. Song, "Improving diffusion inverse problem solving with decoupled noise annealing," *arXiv preprint arXiv:2407.01521*, 2024.

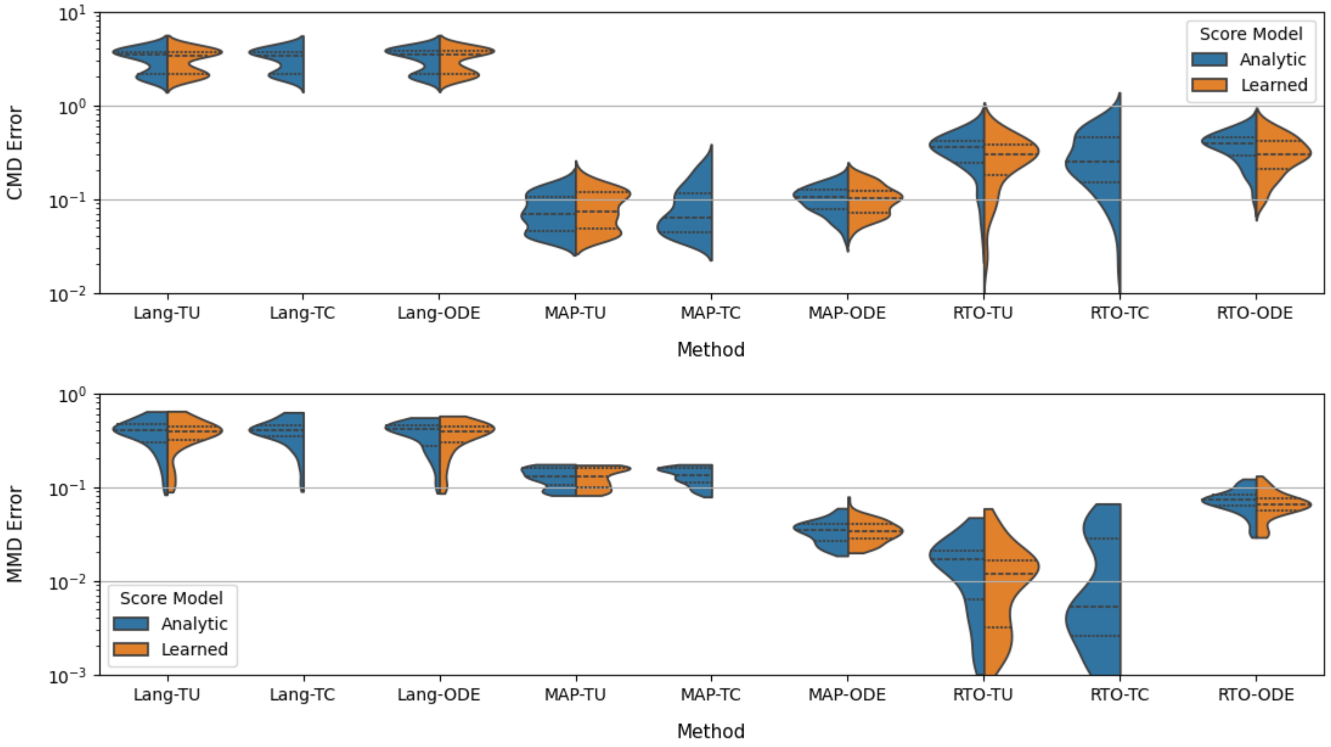

FIGURE S2. **Stylized inpainting study, high noise regime: Two-sided violin plots of the CMD errors (top) and MMD errors (bottom) across the one hundred trials. As can be seen, the 'Lang' variants perform poorly in this problem setting.**

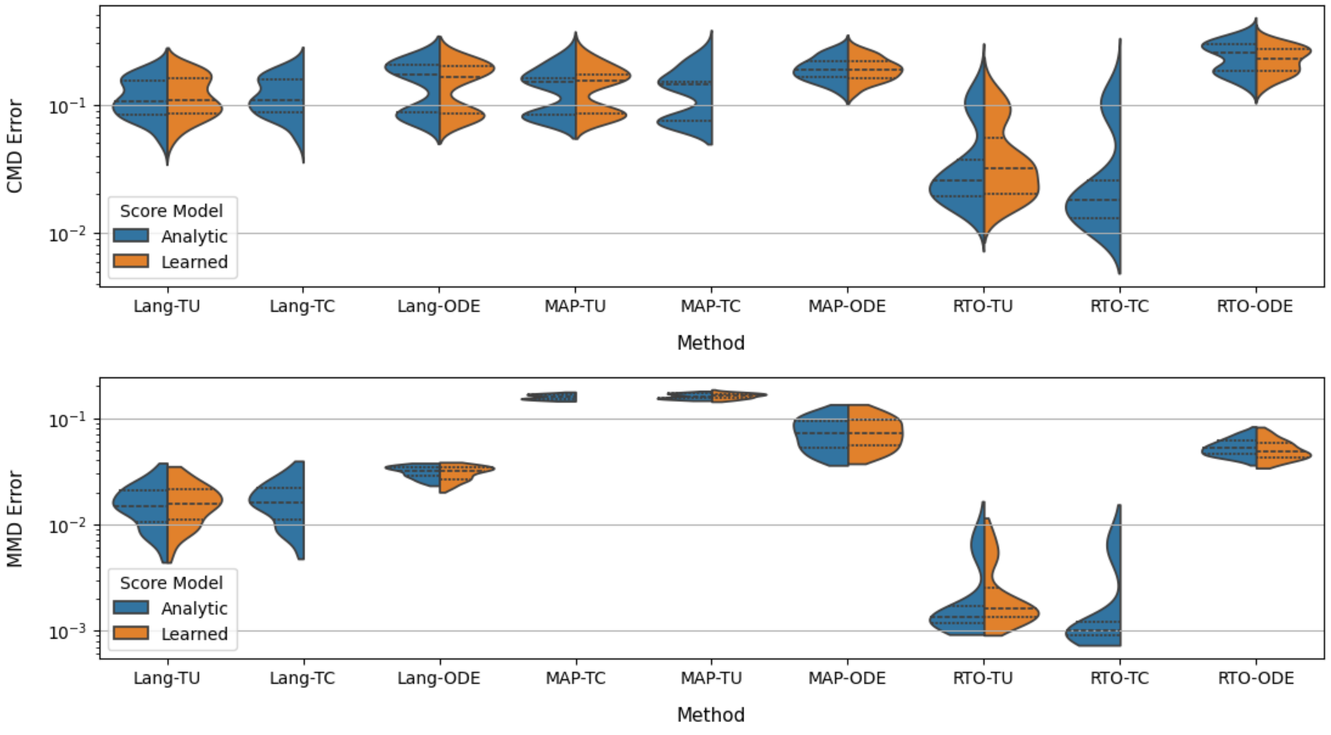

FIGURE S3. **Stylized x-ray tomography study: Two-sided violin plots of the CMD errors (top) and MMD errors (bottom) across the one hundred trials. As can be seen, the 'RTO-TU' and 'RTO-TC' (in analytic score setting) approaches achieve the best performance of the algorithms tested with respect to both metrics.**

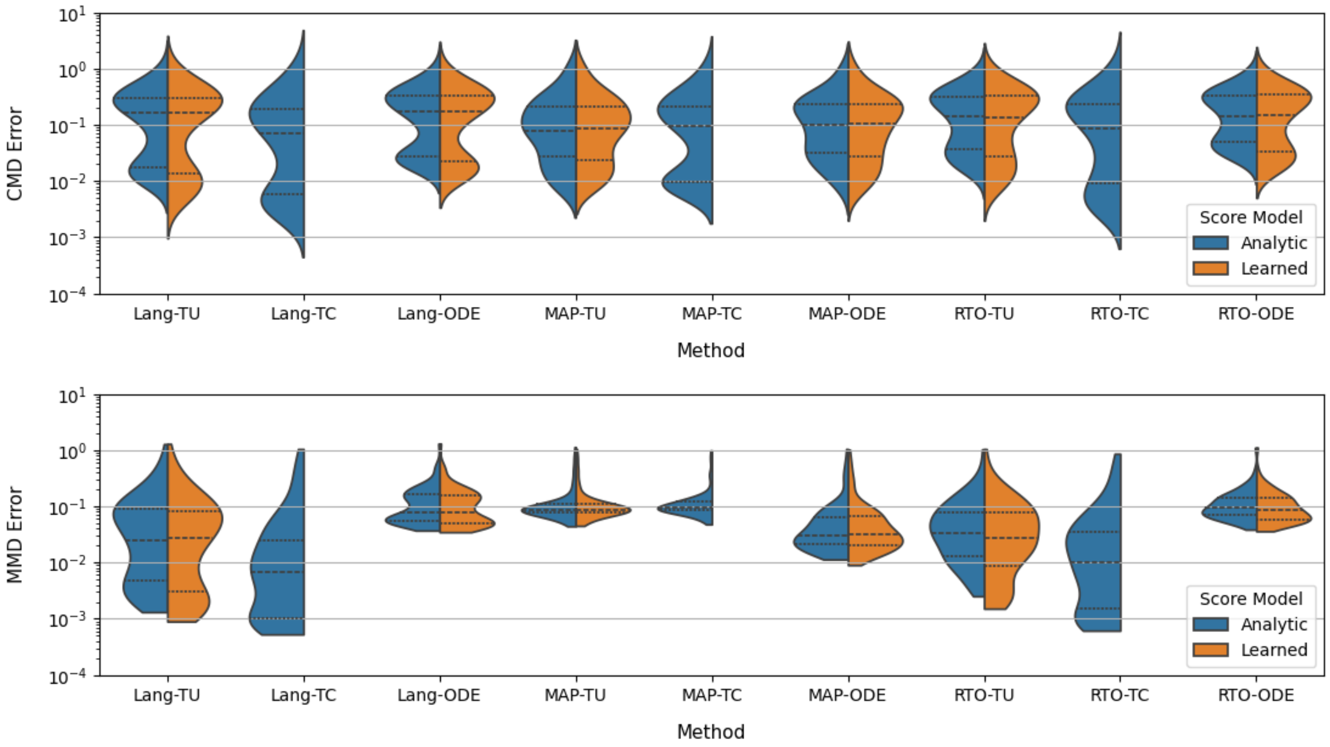

FIGURE S4. Stylized phase retrieval study: Two-sided violin plots of the CMD errors (top) and MMD errors (bottom) across the one hundred trials. As can be seen, the performance of all of the BIPSDA algorithms we tested varies significantly across the trials, with none of the algorithms providing consistently strong performance.

Lang-TU	Lang-ODE	MAP-TU	MAP-ODE	RTO-TU	RTO-ODE
293.2188 (0.1665)	996.1410 (0.3125)	**246.2827 (0.1702)**	949.5303 (0.1598)	246.4814 (0.0713)	949.6074 (0.3833)

TABLE S1. Image inpainting study: Computational time (in seconds) to generate 100 posterior samples (averaged over 10 trials) with different BIPSDA methods. The standard deviation of the runtime is also displayed in parentheses. All experiments were run on a Nvidia A100 GPU with 80 GB of memory. As can be seen, for this problem, in which the cost of approximating the denoising distribution is dominant, the 'TU' BIPSDA algorithm variants are significantly faster than the 'ODE' variants.

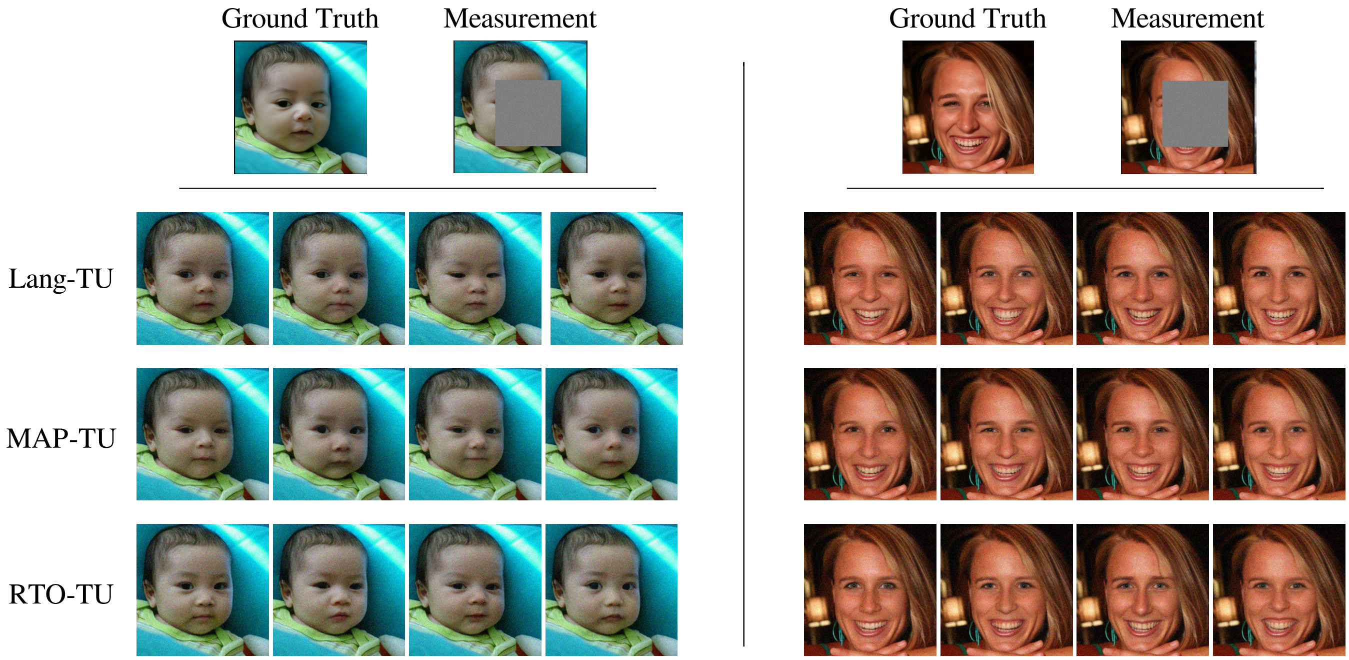

FIGURE S5. Image inpainting study: Uncurated samples from the 'Lang-TU', 'MAP-TU', and 'RTO-TU' BIPSDA algorithms for two different ground-truth images. As can be seen, all three algorithms perform similarly and are capable of generating high-quality and diverse samples, although it is difficult to rigorously validate the quality of the samples due to the lack of analytic ground truth prior in this problem setting.
